# Towards gender equity in Australian health and medical research funding

**DOI:** 10.5694/mja2.51767

**Published:** 2022-11-13

**Authors:** Anne Kelso

**Affiliations:** ^1^ Chief Executive Officer National Health and Medical Research Council Canberra ACT

**Keywords:** Peer review

From 2023, the National Health and Medical Research Council aims to award equal numbers of Investigator Grants to women and men

Gender equity is in the news. The Jobs and Skills Summit in September 2022 and recent reports on women's representation in science, technology, engineering and mathematics (STEM), business leadership and the wider workforce^1^, ^2^, ^3^ have observed Australia's slow progress towards workplace equity and equality for women and men.

This is also true in Australian health and medical research. Although women have enrolled in health and science degrees and completed postgraduate study in large numbers for decades,^4^ these numbers are still not reflected at the highest levels of the research sector.^5^ Men continue to predominate among senior researchers and academic leaders in universities, medical research institutes and hospitals. Men continue to apply for and receive more National Health and Medical Research Council (NHMRC) grants and total funding than women.^6^ Here and elsewhere, men continue to receive more recognition through prizes, conference invitations and other measures of esteem.^7^, ^8^, ^9^

The immediate issue is attrition — the inexorable disappearance of women from the sector with increasing seniority.^5^ The largest grants and the most prestigious awards naturally go to those with a long history of high research productivity and visibility. However, the attrition of women in turn reflects complex factors that have not yet been overcome by the many gender equity initiatives introduced across the sector. These factors range from the legacy effect of past social structures to the practical challenges of combining a research career with family responsibilities, and the explicit and implicit biases that can affect women's opportunities for professional advancement.

## Gender equity in NHMRC funding

NHMRC has been publishing grant outcomes by gender (male, female, indeterminate/intersex or not stated)^10^ for many years and implementing initiatives to improve gender equity in our grant program.^11^ On average across all NHMRC schemes, funded rates (grants as a proportion of applications) have been close to parity for women and men since 2017.^6^ An important contributor to this achievement has been a special measure under the *Sex Discrimination Act 1984* ^12^ to fund additional grants led by women whose application scores fell just below the funding cut‐off (structural priority funding).^11^

Yet the impact of women's attrition persists. This is seen most starkly in the large Investigator Grant scheme, a career stage‐based scheme where women predominate at the earliest postdoctoral stage (Emerging Leadership Level 1) but make up only about 20% of the most senior applicants (Leadership Level 3). The direct consequence of this imbalance is that about 35% more grants and 67% more total funding were awarded to men than women in the first three years of the scheme (2019–2021), despite funding of additional women through NHMRC's structural priority budget.^6^

NHMRC's view is that these disparities are due to the systemic disadvantage experienced by women in the health and medical research sector. They cannot be resolved through individual adjustments of track record scores but require systemic intervention.

NHMRC has little information about researchers who do not identify as either male or female (here referred to as non‐binary) but other evidence suggests they are also likely to be disadvantaged.^13^

## NHMRC's consultation on options to improve gender equity

In 2022, NHMRC has consulted widely on what to do now. Following publication of a CEO communique^6^ in February and public webinars with researchers in February–March 2022, we released a discussion paper^14^ and an online survey in July presenting four options for direct intervention to reduce the gender gap in Investigator Grant outcomes. Roundtables with peak bodies and open forums with researchers around the country were held in July–August to discuss the options and their potential implications for funding outcomes.

Two of the options put forward involved increasing structural priority funding for women from 8% to 20% of the Investigator Grants budget (in the second option, also awarding a fixed research support package to all Leadership Fellows, rather than the current graded package determined by application score). The other two options were to run separate competitions for women and men, then award either equal numbers of grants or equal total funding. Modelling of each option based on past outcomes showed that the additional funded applications would be highly meritorious, scoring above 5 (excellent) or 5.5 (outstanding) on NHMRC's 7‐point scale.^14^

NHMRC proposed that non‐binary applicants be eligible for inclusion in any new gender equity initiative. NHMRC currently lacks evidence to support other approaches but changes to gender data collection from 2023 will provide a basis for further consideration.^14^

Among those who participated in the consultation, there was overwhelming support for a new intervention to improve gender equity in the scheme.^15^ Furthermore, participants favoured separate competitions, particularly the award of equal total funding to women and men, and immediate implementation. There was also support for focusing the intervention on the Leadership levels where gender disparities have been most apparent.

## NHMRC's new special measure

On 12 October 2022, the Minister for Health and Aged Care, the Hon Mark Butler MP, and the Minister for Finance and Minister for Women, Senator the Hon Katy Gallagher, announced that NHMRC would implement a new targeted measure to address gender disparities at the Leadership levels of the Investigator Grant scheme (Box). NHMRC will run separate competitions for women and men with the aim of awarding equal numbers of Leadership grants to the two groups. Structural priority funding will continue to be used to support additional women at Emerging Leadership levels if required to meet gender equity targets. Non‐binary researchers will be eligible for support in both measures.

Box 1 **A new funding framework for the NHMRC Investigator Grant scheme**

**Table:** 

**Career stage**	**Gender equity intervention**	**New initiative?**
Emerging Leadership Levels 1 and 2	Structural priority funding as required	No
Leadership Levels 1–3	Equal grant numbers awarded to female/non‐binary and male applicants	Yes
	A single research support package of $400 000 per annum*	Yes

* Together with equal grant numbers, this change will ensure that total funding awarded to female/non‐binary and male applicants will be similar.

These decisions were bolstered by the 2022 Investigator Grant outcomes^16^ that were being finalised by NHMRC during the national consultation in July–August. On the advice of the NHMRC Research Committee, the structural priority budget was increased to 20% of funds available for Investigator Grants in 2022. However, no intervention was required at Emerging Leadership Level 1 and little at Emerging Leadership Level 2 to achieve comparable numbers of grants and overall funding to women and men. This is encouraging progress that we hope will lead to higher numbers of women applying at the next level when these grants have run their course. A significant gender gap nevertheless remained at Leadership levels; although the additional structural priority funding helped to bridge this gap, it was insufficient to achieve similar grant numbers and overall funding for women and men in the 2022 round. The new funding framework will ensure that this goal is reached in 2023.

The intervention to award equal numbers of Leadership grants to women and men is a new special measure under the *Sex Discrimination Act 1984*.^12^ Its goals are defined, its effectiveness will be monitored annually, and it will cease if it is found to be ineffective or to have achieved its goals.

To our knowledge, this is the first time that a national funding agency has introduced such an intervention for a major grant scheme. Like NHMRC, other funders have implemented various initiatives to foster gender equity in grant funding, including retaining a proportion of a scheme's budget to equalise funded rates for female applicants if required,^17^ similar to our existing structural priority measure.

## Why now?

Why would NHMRC take such a step now? This is unquestionably a big step that will be received well by some and not by others in the health and medical research sector. It also comes at a time of intense pressure on the sector because of low funded rates for most NHMRC grant schemes and disruption of research projects and careers by the COVID‐19 pandemic.

The impact of COVID‐19 is one reason that intervention to reduce gender inequities in the NHMRC grant program is critical now. The pandemic has exacerbated pre‐existing gender inequities in the STEM workforce, with women especially disadvantaged by lockdowns, overseeing children's schooling at home, caring for other family members, and being more likely to be in precarious employment.^18^ Women have been disproportionately represented among the thousands who have lost jobs from universities.^19^ The effects on the shape of the Australian research workforce are likely to be significant and long lasting.

We knew before the pandemic that we could not afford to lose so many women if we are to build the strong and diverse research sector needed to meet our current and future health challenges.^20^, ^21^ Today, we cannot afford to go backwards in our journey to gender equity, as we most certainly will without action.

The changes we have announced will not solve the problem for the whole sector, nor will they address all the challenges we face in supporting a strong research system, but they are significant. As long as intervention is required to achieve gender equity in the Investigator Grant scheme, the new funding framework will ensure that more women have the opportunity to contribute to the improvement of human health through their research. More women will be able to advance their careers. More women will be the visible face of our national research effort, encouraging the next generation of women and men to pursue this most significant and rewarding of career paths.

## Competing interests

No relevant disclosures.

## Provenance

Not commissioned; not externally peer reviewed.
